# Double-J stent versus percutaneous nephrostomy for emergency upper urinary tract decompression

**DOI:** 10.25122/jml-2022-0334

**Published:** 2023-05

**Authors:** Cosmin Cozma, Dragoş Georgescu, Răzvan Popescu, Bogdan Geavlete, Petrişor Geavlete

**Affiliations:** 1. Department of Urology, Faculty of Medicine, Carol Davila University of Medicine and Pharmacy, Bucharest, Romania; 2. Department of Urology, Sf. Ioan Emergency Clinical Hospital, Bucharest, Romania

**Keywords:** double-J stent, percutaneous nephrostomy, urinary drainage

## Abstract

Urinary tract obstruction is a serious condition that can cause significant morbidity in patients with acute obstructive uropathy. Prompt urinary diversion is necessary to prevent further damage to the kidneys. Retrograde ureteral stenting (RUS) and percutaneous nephrostomy (PCN) are the two main treatment options for this condition in many hospitals. This study aimed to compare the effectiveness and safety of PCN and RUS for treating acute obstructive uropathy. We conducted a retrospective study of 1500 consecutive patients who presented to the emergency room between January 2017 and December 2021 and underwent either double-J stenting or percutaneous nephrostomy. Patient characteristics and anatomic data were evaluated using abdominal ultrasonography, computed tomography, blood tests, and/or KUB radiography. Out of the 1500 patients, 1172 patients underwent double-J stenting, while 328 patients received percutaneous nephrostomy initially. In 54 cases where double-J stenting was inefficient, subsequent percutaneous nephrostomy was performed. The majority of cases were efficiently treated with double-J stenting. Double-J stenting was an effective method of urinary drainage in most cases of acute obstructive uropathy.

## INTRODUCTION

Obstruction of the upper urinary tract can result from various conditions. Prompt intervention is required to repair the anatomical defect, remove an acquired blockage, and restore kidney function. Urolithiasis is a common condition that can significantly lower patients' quality of life, with the most life-threatening consequence being septic episodes secondary to an infected blocked system or stone manipulation. Urosepsis, resulting from ureteral stone-induced infection of the hydronephrosis, can lead to sepsis, septic shock, and even death. Immediate decompression of the pelvicalyceal system (PCS) and prompt initiation of antibiotic therapy are crucial life-saving procedures in such cases. If left untreated, total obstruction of the renal drainage system may result in loss of kidney function [1]. Rarely, unilateral or bilateral total urine blockage endangers one kidney's ability to function normally. In these situations, clearing the obstruction also stops the loss of kidney units. Furthermore, renal colic may require rapid decongestion to relieve persistent discomfort.

Percutaneous nephrostomy (PN) and double-J stent (DJS) ureteral catheter placement are emergency procedures used to decompress the PCS [2]. However, the choice of approach is a topic of intense debate. After the infection or sepsis has subsided, the ultimate stone treatment is advised by the European Association of Urology (EAU) recommendations for lithiasis. Primary ureteroscopy (URS) plays an increasingly significant role in treating non-infectious ureteric stones. In addition to DJS and PN, URS is a procedure that urologists are trained to perform [3,4]. Specialized radiologists may also perform PN as long as they are appropriately trained [5]. The choice between employing DJS or PN to relieve pressure in the renal collecting system relies on multiple factors. These can range from the unique circumstances of the clinical case to the competency and practical knowledge of the attending physician.

Another condition that necessitates PCS decompression is malignant obstructive uropathy, which results from secondary extrinsic compression or infiltration of the ureter. Urologists are typically consulted to determine the most suitable form of urinary diversion while considering disease and patient features. Since many of these patients receive continuing treatment, urologists work to remove urinary blockages, alleviate symptoms, and enhance kidney function while maintaining the patient's quality of life and possibly extending overall survival. However, when the retroperitoneal or pelvic disease is advanced, and there is a significant failure rate, such as in the case of pelvic cancers, the endoscopic technique may be technically challenging and even impossible. The success of retrograde ureteral stenting in patients with pelvic malignancy is typically significantly lower in those with extrinsic ureteral obstruction compared to those with internal ureteral obstruction, likely due to the non-progression of the hydrophilic guide and non-identification of the ureteral meatus [6].

On the other hand, the percutaneous technique, which is more intrusive and frequently linked to a higher incidence of infection, bleeding, discomfort, and unintentional tube displacement, may have a detrimental impact on the patient's quality of life. Frail patients may be less willing to accept long-term nephrostomy tubes that require routine replacement due to a decrease in quality of life. Therefore, when assessing the patient's clinical state and life expectancy, the choice of approach must take into account the physician's experience and the patient's preferences. While temporary urine diversion is a common approach in the management of acute urinary obstruction, there is currently no consensus or clear recommendations on the optimal course of action for malignant ureteral obstruction. Regarding obstructive decompression, there is an ongoing debate about the effectiveness of retrograde ureteral stenting (RUS) versus percutaneous nephrostomy (PCN). The aim of this study was to compare the effectiveness and safety of PCN and RUS in the management of acute obstructive uropathy.

## MATERIAL AND METHODS

**Figure 1.**
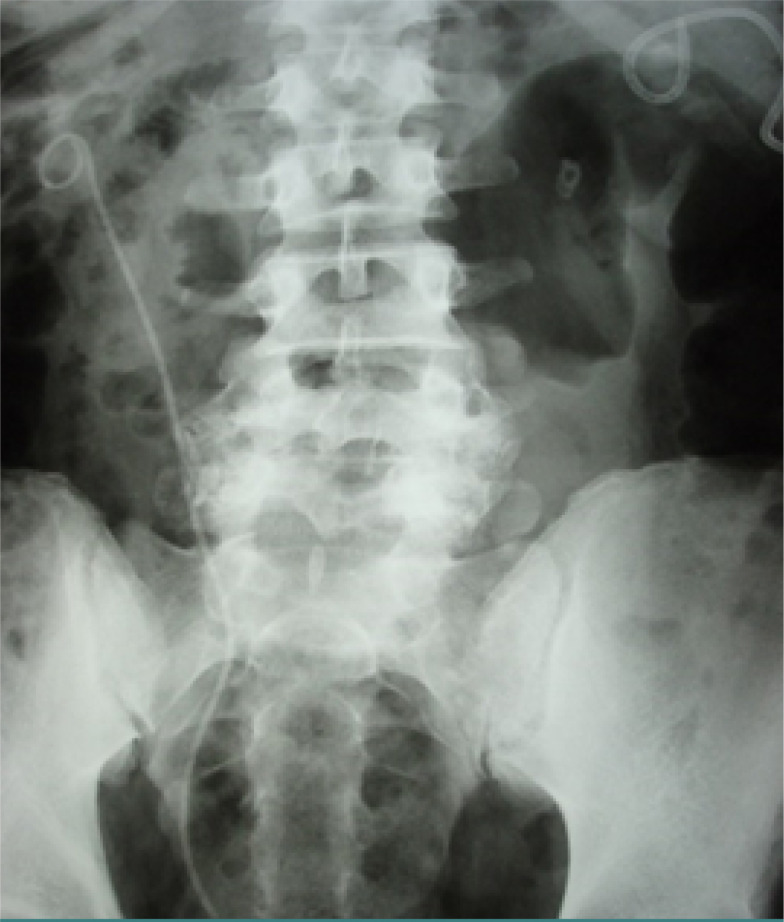
Percutaneous nephrostomy and double-J stent placement performed on the same patient

We conducted a retrospective study over a period of 5 years (January 2017 – December 2021), including 1500 consecutive patients who presented to the emergency room and underwent either double-J stenting or percutaneous nephrostomy (Fig. 1). Patients’ demographic characteristics, clinical features, and anatomical data were observed based on abdominal ultrasonography, computed tomography, blood tests and/or KUB radiography (Table 1).

**Table 1.** Demographic and clinical characteristics

Treatment group N=10	Double-J stent	Percutaneous nephrostomy
Age(years)	59.4	67.1
Gender		
Male	366	136
Female	715	192
**Factors causing obstruction**		
Non-malignant ureteral obstruction	921 (78.5%)	52 (15.85%)
Malignant ureteral obstruction	251 (21.4%)	276 (84.14%)
**Hydronephrosis grade**		
Grade I	211 (18%)	16 (4.87%)
Grade II	507 (43.25%)	83 (25.30%)
Grade III	281 (23.97%)	133 (40.54%)
Grade IV	173 (14.76%)	96 (29.26%)

In this study, we enrolled patients requiring emergency drainage due to either ureteral stones or malignant ureteral obstruction. A urologist decided whether the patient required emergency drainage. The inclusion criteria were patients diagnosed with upper urinary tract stones and urosepsis upon admission or patients with malignant tumors with radiological evaluations resulting from urinary stasis, often with worsening kidney function due to ureteral obstruction. Upper urinary tract stones were identified based on the patient's clinical symptoms, plain radiography, ultrasonography, and CT scans. Urosepsis was identified based on the Sepsis-3.0 criteria as an increase of two or more points in the Sequential Organ Failure Assessment (SOFA) score. Patients with urinary diversion, pregnancy, upper urinary tract urothelial cell cancer, severe sepsis (diagnosed as bacterial septic shock), septic shock, or those unable or unwilling to adhere to the trial's follow-up procedures were excluded from the study.

To determine if a patient had progressed to urosepsis, vital signs (heart rate (HR), breathing rate, and temperature), blood pressure (BP), oxygen saturation (SpO2), degree of consciousness, urine volume and frequency over 18 hours, and skin color and filling were monitored. Microbiological tests, including urine culture and biochemical tests, including White Blood Cell (WBC) count, C-reactive protein (CRP), serum lactate, thrombocyte count, total bilirubin, and arterial blood gas, were also used as diagnostic tools. The SOFA score was calculated by deducting the baseline value to determine the SOFA score change.

Since Escherichia coli is the most frequent pathogenic bacterium in urosepsis, antibiotic treatment was administered empirically after admission. All patients underwent testing for antimicrobial susceptibility and urine culture before beginning the antibiotic treatment. Following the publication of the drug susceptibility results, our patients received the corresponding sensitive antibiotics. Emergency drainage was performed either by RUS or PCN.

All data were collected from the medical records and analyzed using Microsoft Excel and Word, available on Microsoft Office 18.2008.12711.0 (Microsoft Corp., USA) and IBM SPSS Statistics Version 26 (IBM, USA).

## RESULTS

Data were collected from 1500 patients, comprising 907 (60.46%) females and 502 (39.53%) males. The mean age of the patients was 61.08 years. Percutaneous nephrostomy was performed in 328 patients from the start, whereas double-J stenting was performed in 1172 patients (Figure 1). In 54 cases of double-J stenting, percutaneous nephrostomy was subsequently performed due to the inefficiency of the double-J stent.

**Figure 2.**
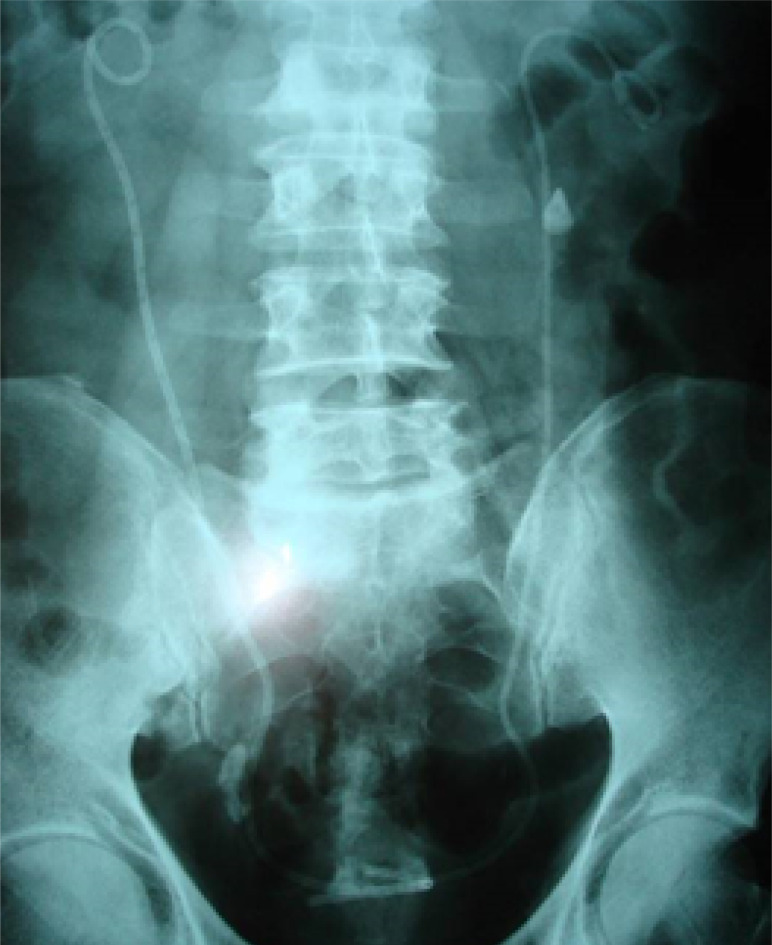
KUB radiography of bilateral double-J stents

Out of the patients who received double-J stenting, 921 presented with urinary tract obstruction due to non-malignant pathologies such as urolithiasis, ureteral strictures, or PUJ stenosis, while 251 patients presented with malignant urinary tract obstruction (Fig. 2). In 30 cases, the double-J stent proved to be ineffective, and the patients required percutaneous nephrostomy instead. In those cases, the patients presented with dilated PCS or elevated nitrogen retention products (serum creatinine and urea) after the placement of the double-J stent.

**Figure 3.**
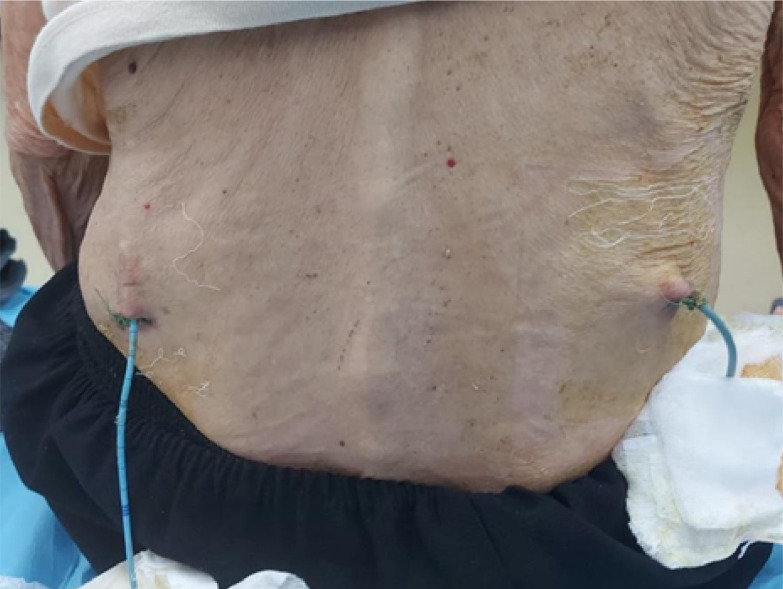
Bilateral percutaneous nephrostomy

Among the patients who underwent percutaneous nephrostomy, 276 presented with malignant ureteral obstruction causing obstructive uropathy, while 52 presented with non-malignant urinary tract obstruction (Fig. 3). In terms of the drainage method, the majority of patients in the percutaneous nephrostomy group received a 10F pigtail catheter, whereas the majority of patients in the double-J stenting group received a 6F stent. Interestingly, 50% of patients who underwent percutaneous double-J stenting were discharged from the hospital in less than 24 hours, compared to only 20% in the percutaneous nephrostomy group.

## DISCUSSION

Urosepsis is a serious condition with a high fatality rate and urinary tract blockage is a significant risk factor for its development. Prompt removal of the blocking cause is crucial in preventing the development of urosepsis and septic shock [7]. Retrograde ureteral stenting and percutaneous nephrostomy are both effective emergency draining techniques, with technical success rates ranging from 82 to 99% [8,9]. Malignant ureteral obstruction is a concerning symptom frequently linked to a poor prognosis, and patients with advanced malignancies are often affected by this condition [10]. The gradual development of ureteral blockage can lead to dull pain, weariness, and lethargy. Therefore, it is crucial to use effective emergency draining techniques to prevent the development of urosepsis and septic shock [11].

Sepsis is known to affect men more often than women, but urosepsis is more frequent in women. In our study, women made up the majority of participants (60.46%). Kumar et al. hypothesized that postmenopausal estrogen shortage, atrophic vaginitis, cleanliness of the perineum, cystitis, and usage of pessaries might all contribute to the greater prevalence of urinary sepsis in women. In a nationwide inpatient sample (NIS) study conducted in the USA, women were found to be twice as likely as men to have urolithiasis in combination with a bacterial infection [12].

Urosepsis can cause a wide range of clinical symptoms and signs, including fever, bladder irritation, low back pain, abdominal discomfort and bloating, gross hematuria, nausea, vomiting, and decreased level of consciousness. Sepsis is typically diagnosed when there are observable signs of infection, coupled with symptoms of organ failure, systemic inflammation, and extended periods of low blood pressure associated with lack of oxygen in the tissues [1]. An increase of two or more points in the SOFA score can indicate the presence of organ dysfunction, which is a crucial factor in determining the severity of urosepsis.

Fever is a common symptom associated with upper urinary tract blockage and is often used as a warning sign for emergency drainage. In this study, all patients with non-malignant urinary tract blockage also had fever symptoms, which improved after emergency drainage. Active treatment against the underlying cause of fever is essential. Prompt decompression of infected urine can result in an immediate increase in renal plasma flow and the amount of antibacterial agents in the renal parenchyma and urine. This, in turn, leads to a decrease in the bacterial burden and debris load in the collection system [13].

C-reactive protein (CRP) is a vital marker for diagnosing urosepsis. Similar to sepsis caused by other factors, the severity of urosepsis largely depends on the patient's response. Urosepsis risk factors include both systemic and local variables, with examples of local causes including urinary blockage and urinary stones. In a study analyzing 143 patients with acute obstructive pyelonephritis caused by upper urinary calculi and hospitalized in four Japanese hospitals, Fukuashi et al. found that diabetes and a CRP level of 10 or higher were significant independent risk factors for septic shock, based on multivariate analysis. Emergency drainage in urosepsis patients led to a significant reduction in CRP in both groups [14]. Among patients in our study with non-malignant tract blockage, 87.21% (791/907) had diabetes.

Effective emergency drainage is crucial in preventing urosepsis from developing into septic shock and leading to mortality. The choice of emergency drainage method for urosepsis should take future surgical treatments into account. Patients who received emergency PCN treatment after sepsis resolution were more likely to receive percutaneous nephrostomy treatment, whereas those chosen for emergency RUS treatment were more likely to have ureteroscopy treatment [15].

Early empiric antibiotic therapy is crucial when sepsis is suspected, and microbiological testing should be conducted to determine the causative bacteria for effective treatment. In this study, the urine culture revealed E. coli as the most common bacterium. Other enterobacteria such as Proteus spp., Enterobacter spp., Klebsiella spp., non-fermenting microorganisms like P. aeruginosa, and Gram-positive microorganisms are also commonly found in urosepsis. However, if the host's defense is compromised, Candida spp. and Pseudomonas spp. may also cause urosepsis [16,17].

Our study found that placing ureteral stents resulted in shorter surgical time and hospital stay than percutaneous nephrostomy, with similar overall complication rates. However, the nephrostomy group had a higher inadvertent displacement rate. There were no significant differences between the two methods in terms of complications or creatinine level decline following decompression. Ureteral stent implantation was found to be more effective in terms of procedural outcomes, efficacy, and handling problems. The endoscopic technique also allowed for quicker patient release and reduced the chance of tube displacement, which is particularly important for elderly patients. The use of ureteral stents may also avoid the need for repeated treatments, which can be challenging for older individuals.

Our study provides two important findings. Firstly, ureteral stenting is a less invasive procedure that patients prefer, leading to a quicker discharge, longer interval between exchanges, and reduced risk of tube displacement. The use of ureteral stents can also prevent the need for repetitive and unnecessary treatments, saving both time and money for other palliative procedures. This aspect is particularly significant. Secondly, percutaneous nephrostomy is not inferior to ureteral stenting as a primary drainage procedure, especially in cases of pelvic malignancy or in patients with shorter life expectancies who only require palliative relief of ureteral obstruction. There were no differences in complication rates or unplanned device replacements observed. However, there are limitations to our study. It is a retrospective study, and we acknowledge that urologist preferences can influence the decision on the best course of treatment. Therefore, further prospective randomized studies are necessary to confirm our preliminary findings.

## CONCLUSION

Although both percutaneous nephrostomy and double-J ureteral stenting are effective techniques for treating acute urinary tract obstruction, our research supports using double-J ureteral stents due to their ease of maintenance. Ureteral stent placement should be recommended whenever possible. However, in cases where malignant obstruction prevents the insertion of a JJ stent, percutaneous nephrostomy remains a safe alternative.
